# β-NiS modified CdS nanowires for photocatalytic H_2_ evolution with exceptionally high efficiency†
†Electronic supplementary information (ESI) available: SEM, TEM, and EDX examinations, XRD identification, and photocatalytic evaluation of additional samples. Comparison of the photocatalytic performances of photocatalysts reported in the literature. See DOI: 10.1039/c7sc03928j


**DOI:** 10.1039/c7sc03928j

**Published:** 2017-12-13

**Authors:** Shundong Guan, Xiuli Fu, Yu Zhang, Zhijian Peng

**Affiliations:** a State Key Laboratory of Information Photonics and Optical Communications , School of Science , Beijing University of Posts and Telecommunications , Beijing 100876 , P. R. China . Email: xiulifu@bupt.edu.cn ; Fax: +86-10-62282054 ; Tel: +86-10-62282452; b School of Science , China University of Geosciences , Beijing 100083 , PR China . Email: pengzhijian@cugb.edu.cn ; Fax: +86-10-82322624 ; Tel: +86-10-82320255; c School of Engineering and Technology , China University of Geosciences , Beijing 100083 , PR China

## Abstract

Co-catalysis is regarded as a promising strategy to improve the hydrogen evolution performance of semiconductor-based photocatalysts. But developing a simple and effective technique to achieve the optimal synergy between co-catalysts and host photocatalysts has been a great challenge. Herein, hybrid photocatalysts consisting of β-NiS modified CdS nanowires (NiS/CdS NWs) have been synthesized *via* a simple and green hydrothermal route using CdS NWs as the template from thiourea and nickel acetate in the presence of sodium hypophosphite. As a result, a metal Ni intermediate was formed *via* an electroless plating process assisted by H_2_PO_2_^–^, which facilitated the growth of highly conducting flake-like β-NiS nanostructures onto the surface of the CdS NWs. With the optimal loading amount of NiS, the obtained NiS/CdS NWs present a record-high photocatalytic activity for H_2_ evolution in lactic acid aqueous solutions under visible light irradiation. At 25 °C, the rate of H_2_ evolution was measured as 793.6 μmol h^–1^ (over a 5 mg photocatalyst sample), which is nearly 250-fold higher than that over pure CdS NWs, and the apparent quantum yield reached an exceptionally high value of 74.1% at 420 nm. The mechanism for the photocatalytic H_2_ evolution over the present NiS/CdS NWs was also proposed. This strategy would provide new insight into the design and development of high-performance heterostructured photocatalysts.

## Abstract 2

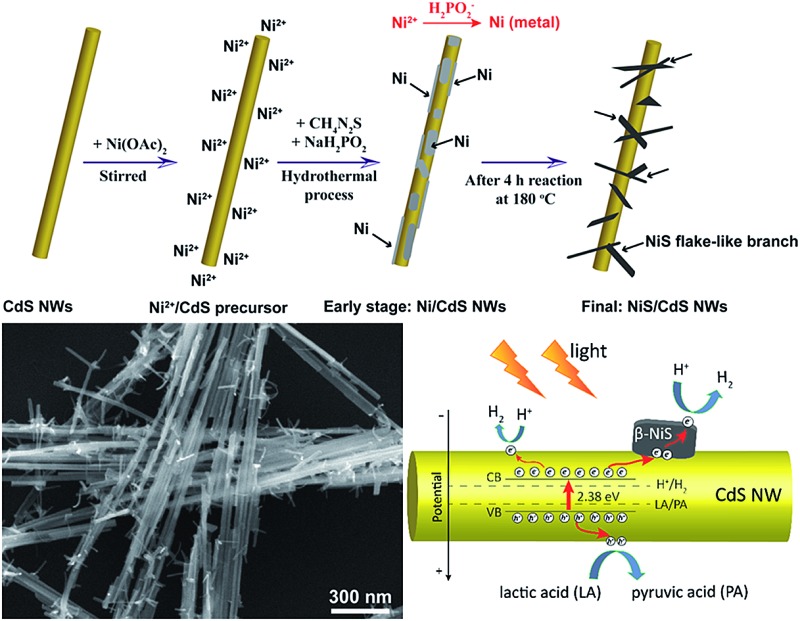
Synthesis of an exceptionally highly efficient NiS–CdS hybrid photocatalyst for H_2_ evolution.

## 1 Introduction

To alleviate the ever-increasing consumption of fossil fuels and the associated environmental pollution as well as global climate change, considerable efforts have recently been devoted to developing clean, abundant, and renewable energy as an alternative to fossil fuels. Among the various technologies proposed so far, splitting water to produce hydrogen (H_2_) fuel through sunlight irradiation, during which the solar energy can be converted to storable chemical energy, represents a very promising and sustainable route.^1^–^3^ Since Honda and Fujishima reported the photocatalytic splitting of water over TiO_2_-coated electrodes in 1972,^4^ extensive investigations have been performed on developing various semiconductor-type photocatalysts. However, it remains a great challenge to obtain highly active, low-cost, and non-toxic photocatalysts capable of catalyzing water splitting under the irradiation of visible light. To improve the photocatalytic H_2_ evolution reaction (HER) activity, loading a co-catalyst onto the host semiconductor photocatalyst has proven to be an effective strategy, which can dramatically promote the separation of photo-excited charges, and lower the activation energy or overpotential for the reactions.^5^,^6^ For example, by introducing a noble metal as the co-catalyst (*e.g.* Pt 7,8 or Au 9), the photocatalytic H_2_ production efficiency becomes much higher than that achieved with bare photocatalysts. Nonetheless, the high cost of noble metals hampers their applications as co-catalysts in practical photocatalytic reactors. Therefore, it is of utmost importance to develop a low-cost, highly efficient noble metal-free photocatalytic HER system.

In particular, in the last decade, cadmium sulfide (CdS) has received considerable attention for use as an efficient HER photocatalyst due to its suitable direct band-gap (∼2.5 eV) allowing for the absorption of visible light of the solar spectrum, as well as its appropriate conduction band (CB) and valence band (VB) positions, which are thermodynamically favorable for water splitting.^10^ However, pure CdS does not possess high efficiency for H_2_ production because of the heavy photo-corrosion under photoelectrochemical conditions.^11^ Hence, many attempts have been made to modify CdS in order to improve its photocatalytic performance. Specifically, it has been demonstrated that many co-catalysts based on earth-abundant transition metal elements including molybdenum (Mo),^12^–^15^ tungsten (W),^16^–^18^ cobalt (Co),^19^–^22^ nickel (Ni),^3^,^5^,^23^–^31^ and copper (Cu),^32^,^33^ and their corresponding chalcogenides, oxides, and phosphides, could be used in combination with CdS to significantly enhance the photocatalytic efficiency for the HER. Among them, the low band-gap semiconductor, nickel sulfide (NiS), has been considered as a promising alternative to Pt due to its ease of fabrication, low-cost, high power conversion efficiency, high electrical conductivity, and most importantly, friendliness to the environment.^34^ In the literature, Zhang *et al.* first prepared a NiS/CdS nanoparticle (NP) hybrid photocatalyst for the HER *via* a simple hydrothermal loading method, and it showed a high apparent quantum yield (AQY) of 51.3% at 420 nm at room temperature.^28^ Later on, Qin *et al.* proposed a one-step hydrothermal approach to synthesize NiS/CdS NPs with an enhanced AQY for the photocatalytic HER up to 60.4% at 420 nm at 35 °C, which was ascribed to the optimized contact between the co-catalyst and host photocatalyst.^30^

Notwithstanding the remarkable progress, the efficiency of the NiS–CdS composite catalysts reported so far remains unsatisfactory because: (1) CdS and NiS are typical n-type and p-type semiconductors, respectively. It is easy for them to form a p–n junction when they hybridize with each other, which could effectively reduce the recombination rates of photo-generated electrons and holes, thus dramatically enhancing the photocatalytic activity.^29^ However, in such a p–n junction structure, the photo-generated electrons from CdS cannot be transferred to NiS due to the presence of a built-in electric field from CdS to NiS. As a photocatalyst, CdS in such a composite serves as the active sites of H^+^ reduction (which is beneficial for hydrogen production), while NiS behaves as the oxidation active sites. In this case, the high electrocatalytic HER activity of NiS commonly observed cannot be expected to contribute to photocatalytic hydrogen production. As a result, the photocatalytic HER performance of such NiS–CdS composite catalysts could only be enhanced to a limited degree;^29^ (2) although β-NiS has been proven to have higher electrocatalytic activity for hydrogen production than other polymorphs of nickel sulfides due to its higher electrical conductivity, previously reported NiS co-catalyst materials always comprise a mixture of multiple phases with α-NiS and/or nickel polysulfides as the major components, and contain no or very little β-NiS. This is because of the difficulty in synthesizing pure β-NiS *via* the existing hydrothermal methods;^35^ (3) the photocatalytic activity is also affected by the morphology of the catalysts. In particular, one-dimensional (1D) nanostructures (*e.g.* nanowires and nanotubes) offer several advantages, for example, a large surface area resulting from their high aspect ratios, high charge separation and transfer efficiencies, and enhanced light absorption ability, which could substantially improve the activity of the photocatalytic HER.^3^,^36^,^37^ However, the challenge lies in obtaining good contact between the CdS semiconductor host photocatalysts and co-catalysts over the entire 1D structure to enhance the transfer efficiency of photo-generated carriers.

**Fig. 1:**
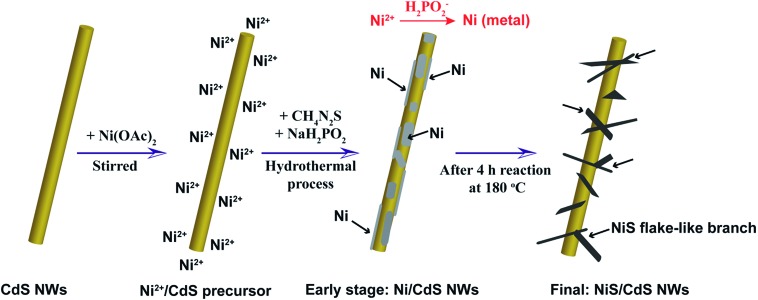
Schematic diagram of the formation process for the present NiS/CdS NWs.

To address these problems, we herein develop a simple and green hydrothermal synthesis route to fabricate a β-NiS modified CdS nanowire (NiS/CdS NW) hybrid photocatalyst using CdS NWs as the scaffold. Our strategy is schematically illustrated in Fig. 1. During the formation of the NiS/CdS hybrid structure, sodium hypophosphite (NaH_2_PO_2_) was used as the reducing agent to assist the growth of the NiS co-catalyst through an electrolessly plated Ni film intermediate. As a result, almost pure β-NiS nanoflakes can be obtained, and these are highly dispersed and well adhered onto the surface of the CdS NWs, resulting in a large contact area between the co-catalyst NiS nanoflakes and host CdS NWs. Due to the high electrical conductivity of β-NiS, fast charge separation between photo-generated electron–hole pairs and high carrier transfer efficiency were realized, which were able to greatly enhance the photoelectric conversion efficiency of the composite catalyst. Besides, such an electroless plating process could effectively retain the formation of a p–n junction between CdS and β-NiS in this hybrid structure. In this case, the highly electrocatalytically active β-NiS would also serve as the active sites for photocatalytic H_2_ production, because the photo-generated electrons from the CdS NWs could be easily transferred to β-NiS, namely, the reduction of H^+^ to H_2_ can also proceed on the surface of β-NiS, thus maximizing the photocatalytic HER activity of the composite catalyst. By optimizing the loading of NiS, the NiS/CdS NW hybrid catalysts can achieve a record-high photocatalytic activity among all the CdS-based noble metal-free photocatalysts for H_2_ evolution under visible light irradiation (*λ* ≥ 420 nm). The rate of H_2_ evolution was measured to be 592.1 μmol h^–1^ (over a 5 mg photocatalyst sample) at 7 °C, and the AQY was 57.8% at 420 nm. When the reaction temperature was maintained at 25 °C, the yield and AQY were further increased up to 793.6 μmol h^–1^ and 74.1%, respectively. Such high photocatalytic activity can be attributed surely to the optimized synergistic effect between the highly reactive β-NiS and CdS NWs. In addition, this simple, cost-effective, and environmentally friendly strategy would provide new insight into the design and development of high-performance heterostructured photocatalysts.

## 2 Experimental

### 2.1 Chemicals and materials

Cadmium acetate dihydrate (Cd(CH_3_COO)_2_·2H_2_O, 98.0+%), sulfur powder (S, 99.5%), nickel acetate tetrahydrate (Ni(CH_3_COO)_2_·4H_2_O, 98.0+%), sodium hypophosphite monohydrate (NaH_2_PO_2_·H_2_O, 98.0–103.0%), lactic acid (C_3_H_6_O_3_, 85.0+%), and sodium sulfite anhydrous (Na_2_SO_3_, 97.0+%) were purchased from Sinopharm Chemical Reagent Co., Ltd (Shanghai, China). Thiourea (CH_4_N_2_S, 99.0+%) and sodium sulfide nonahydrate (Na_2_S·9H_2_O, 98.0+%) were bought from Xilong Scientific Co., Ltd (Shantou, China). Ethylenediamine (C_2_H_8_N_2_, 99.0+%) was purchased from Tianjin Fuchen Chemical Reagents (Tianjin, China). All chemicals were used as received without further purification.

### 2.2 Preparation of CdS NWs

CdS NWs were fabricated according to a previous report with some modifications.^38^ Typically, 0.2665 g of Cd(CH_3_COO)_2_·2H_2_O and 0.0641 g of sulfur were dispersed in 40 mL of ethylenediamine under vigorous stirring and then transferred into a 50 mL Teflon-lined autoclave. The autoclave was heated up to 200 °C, maintained at this temperature for 20 h, and then cooled down naturally to room temperature. Finally, the resultant precipitate (CdS NWs) was washed and then dispersed in deionized water for use.

### 2.3 Preparation of NiS/CdS NWs and NiS nanostructures

To synthesize the proposed NiS/CdS NW hybrid photocatalyst, 29 mg (∼0.2 mmol) of CdS NWs (aqueous suspension, where the concentration was estimated on the basis of dry powders) and a designated amount of Ni(CH_3_COO)_2_·4H_2_O were dispersed in 50 mL of deionized water, and stirred for 3 h. Then, an appropriate amount of thiourea in a Ni/S feed molar ratio (FMR) of 1 : 4 and 0.6 mmol of NaH_2_PO_2_·H_2_O were added into the solution and kept stirring vigorously. Subsequently, the mixture was transferred to a 100 mL Teflon-lined autoclave and solvothermally treated at 180 °C for 4 h, for which it was heated from room temperature to 180 °C in *ca.* 40 min. After the autoclave was cooled down naturally to room temperature, the precipitate was collected and washed with distilled water and ethanol respectively two times. Finally, the product was dispersed in ethanol for use. In this work, the NiS loading was adjusted by changing the Ni/Cd FMR in the range of 0.2 to 1.2 for the reactions, while all the other conditions were kept unchanged.

As for the pure NiS nanostructures, they were synthesized by a similar hydrothermal reaction without the presence of CdS NWs.

### 2.4 Materials characterization

The phase composition of the obtained products was identified *via* grazing incidence X-ray diffraction (GI-XRD, D/max-RB, Japan; Cu Kα radiation, *λ* = 1.5418 Å) in a continuous scanning mode with a scanning rate of 6° min^–1^ and an X-ray incidence angle of 1°. The morphology and structure were examined by field emission scanning electron microscopy (FE-SEM, S4800, Hitachi, Japan), transmission electron microscopy (TEM, Tecnai G2 F20 U-TWIN, FEI, America) and high-resolution TEM (HRTEM). The chemical composition was measured by an energy dispersive X-ray (EDX) spectrometer attached to the TEM instrument. The chemical state of the elements in the samples was investigated by X-ray photoelectron spectroscopy (XPS, Thermo ESCALAB MKII, Thermo VG Scientific Ltd., UK), and the results were calibrated by the C 1s line (binding energy, 284.8 eV). The UV-visible absorption spectra were recorded on a Varian Cary 5000 UV-vis spectrophotometer (Agilent, America).

### 2.5 Photocatalytic H_2_ evolution

Photocatalytic water splitting was carried out in a LabSolar photocatalytic H_2_ evolution system (Perfectlight, Beijing, China) equipped with a 300 W Xe lamp (MICROSOLAR300, Perfectlight, Beijing, China). In a typical photocatalytic reaction, 5 mg of the catalysts was dispersed in an aqueous solution (100 mL) containing 20 vol% lactic acid. After that, the system was sealed and pumped out to a vacuum level of –0.1 MPa. During the reaction, the circulating cooling water system worked so as to keep the reaction at 7 or 25 °C, and the reactor, under magnetic stirring, was irradiated by visible light (*λ* ≥ 420 nm) provided by the 300 W Xe lamp with an UV cut-off filter. The distance between the Xe lamp and the surface of the reaction solution was kept at 20 cm, and the effective irradiation area was measured as 12.57 cm^2^. The gases evolved were analyzed on-line with a gas chromatograph with N_2_ as the carrier gas (GC-7900, Xuansheng Scientific Instrument Co. Ltd, Shanghai, China).

The AQY was calculated according to eqn (1) using a 300 W Xe lamp with a band-pass filter (*λ* = 420 ± 5 nm) and an irradiatometer (FZ-A, Photoelectric Instrument Factory of Beijing Normal University, Beijing, China):1
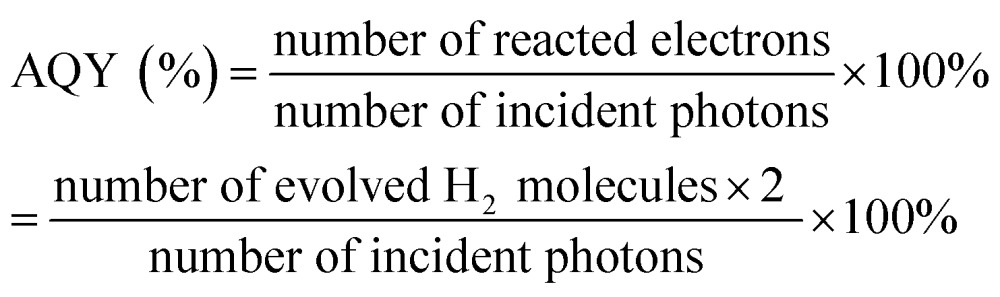



The measured power of the light was 5.52 mW cm^–2^ and the irradiation area was 12.57 cm^2^. So, the corresponding number of incident photons was 1.466 × 10^17^ photons per second.

### 2.6 Photoelectrochemical measurements

#### Electrode fabrication

The working electrode was prepared by dropping a suspension (50 μL) from the samples onto the surface of a fluorine-doped tin oxide (FTO) glass substrate (1.5 × 1.5 cm). Such a suspension was prepared by adding 5 mg of the as-synthesized samples into a mixture containing 20 μL of 5 wt% Nafion solution and 500 μL of absolute ethanol. The working electrodes were dried at room temperature.

#### Transient photocurrent and incident photon-to-electron conversion efficiency (IPCE) tests

Transient photocurrent measurements were performed on a CHI660E electrochemical workstation (Chenhua Instrument, Shanghai, China) in a standard three-electrode system with the as-prepared FTO electrode as the working electrode, a Pt plate as the counter electrode, and a Ag/AgCl electrode (saturated KCl) as the reference electrode. An aqueous solution containing 0.1 M Na_2_S and 0.02 M Na_2_SO_3_ was used as the electrolyte. The system was degassed by high-purity nitrogen for about 30 min before each measurement, but left open to air during the test. A 300 W Xe lamp with an UV cut-off filter was used to provide the visible light (*λ* ≥ 420 nm). The IPCE measurements were performed under the same conditions, in which the monochromatic light irradiation was provided by the same Xe lamp but with different band-pass filters (*λ* = 400 ± 5, 420 ± 5, 480 ± 5, 520 ± 5, 550 ± 5, 600 ± 5, 650 ± 5, and 700 ± 5 nm). All the photo-responsive signals of the samples were measured under chopped light at 0.0 V *vs.* Ag/AgCl.

#### Electrochemical impedance spectroscopy (EIS) and Mott–Schottky (M–S) measurements

The EIS measurements were also carried out with the above-mentioned working electrodes in the CHI660E three-electrode system under the same conditions. During the measurement, the frequency was in the range of 0.01 to 10^5^ Hz, and the AC amplitude was set at 5 mV *vs.* Ag/AgCl. The M–S plots were also recorded using the CHI660E three-electrode system at an AC frequency of 1 kHz, with the amplitude as 5 mV *vs.* Ag/AgCl, but the electrolyte was changed to a neutral aqueous solution containing 0.5 M Na_2_SO_4_. All these experiments were conducted under dark conditions.

## 3 Results and discussion

### 3.1 Photocatalytic performance for H_2_ evolution

**Fig. 2:**
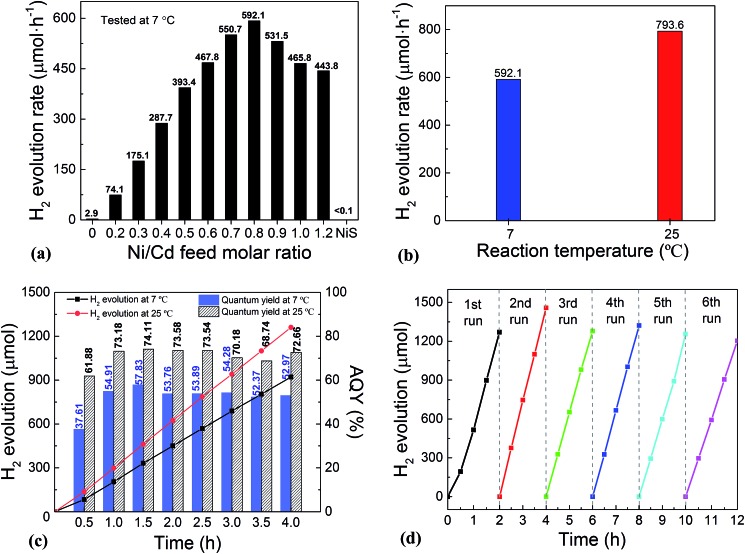
Photocatalytic H_2_ evolution performance of NiS/CdS NWs. (a) H_2_ evolution rate at 7 °C over the NiS/CdS NWs prepared at different Ni/Cd FMRs. (b) H_2_ evolution rate at 7 and 25 °C over the NiS/CdS NWs prepared at an optimal Ni/Cd FMR of 0.8. Both data were calculated based on the amount of H_2_ generated in the first 4 h of the reactions. (c) Time dependence of the H_2_ evolution and AQY at 7 and 25 °C over the optimized NiS/CdS NWs. (d) Cycling runs for H_2_ evolution at 7 °C over the optimized NiS/CdS NWs. Reaction conditions: 5 mg of the catalysts; 100 mL of aqueous solution containing 20 vol% lactic acid; and visible light irradiation provided by a 300 W Xe lamp with an UV cut-off filter (*λ* ≥ 420 nm) for (a), (b), and (d), or a band-pass filter (*λ* = 420 ± 5 nm) for (c).

The photocatalytic performance for H_2_ evolution of the NiS/CdS NWs prepared at different Ni/Cd FMRs was first evaluated under visible light irradiation at 7 °C, and the result is shown in Fig. 2a. For comparison, the performance of pure CdS NWs (*i.e.* NWs prepared at a Ni/Cd FMR of 0 : 1) and NiS nanostructures (*i.e.* those prepared at a Ni/Cd FMR of 1 : 0 *via* a similar hydrothermal process without a CdS template) was also investigated. For the HER, after a series of optimizations, 20 vol% lactic acid was chosen as the sacrificial agent (Fig. S1 and S2, ESI†). As is seen from Fig. 2a, the photocatalytic HER rate of the pure CdS NWs was rather low (2.9 μmol h^–1^), presenting a poor photocatalytic activity as reported in the literature.^3^,^28^ When NiS was loaded onto the CdS NWs, the photocatalytic HER rate was significantly increased. And increasing the loading amount of NiS onto CdS through increasing the Ni/Cd FMR (see the practical molar percentage of NiS for each sample in Table S1†) first led to an increase and then a decrease in the H_2_ evolution rate, with the highest HER rate of 592.1 μmol h^–1^ achieved by the NiS/CdS hybrid photocatalyst prepared at a Ni/Cd FMR of 0.8, which is *ca.* 204-fold higher than that of pure CdS NWs. As for the hybrid photocatalysts with a higher content of NiS (prepared at a Ni/Cd FMR higher than 0.8), the H_2_ evolution rate decreased gradually as the loading of NiS increased, which might be due to the shielding effect of excessive NiS.^29^,^30^ Consequently, in the subsequent experiments, our focus was placed on the optimized photocatalysts prepared at a Ni/Cd FMR of 0.8. In order to examine whether the enhancement in HER activity observed for the NiS/CdS hybrid photocatalyst results merely from NiS, the photocatalytic HER of pure NiS nanostructures was also investigated. As is seen from Fig. 2a, there was no appreciable amount of H_2_ detected when the pure NiS nanostructures were used, revealing that NiS alone was not an active photocatalyst for H_2_ evolution, but served only as a co-catalyst here. Therefore, the enhanced HER activity should be attributed to the synergistic effect between NiS and CdS NWs.

In consideration of the stability of the whole photocatalytic HER and the protection of the gas chromatograph from vapor erosion, most of our tests were conducted at 7 °C stabilized by a circulating cooling water system. But for comparison with literature reports, the photocatalytic HER activity of the optimized NiS/CdS NWs was also investigated at 25 °C. As is shown in Fig. 2b, when the reaction temperature was increased to 25 °C, the photocatalytic HER rate was enhanced up to 793.6 μmol h^–1^, which could be attributed to the easier desorption of both the generated H_2_ and the oxidized lactic acid molecules from the surface of the photocatalyst at a higher temperature.^39^

To further investigate the AQY for the photocatalytic HER, 5 mg of the optimized NiS/CdS NWs was dispersed in 100 mL of aqueous solution containing 20 vol% lactic acid, and then the solution was irradiated by visible light (*λ* = 420 ± 5 nm) provided by a 300 W Xe lamp with a band-pass filter at 7 and 25 °C. As is seen from Fig. 2c, the amount of the generated H_2_ was increased gradually over time, but the AQYs did not vary substantially after 1 h of irradiation, and these were roughly 55% at 7 °C and 73% at 25 °C. However, those values were lower in the first half hour (about 37.61% at 7 °C and 61.88% at 25 °C, respectively), which might be due to an induction period at the early reaction stage and the dissolution of H_2_ in the solution.^3^ The highest AQYs were obtained at 1.5 h at both 7 and 25 °C, reaching 57.83% and 74.11%, respectively, which, to the best of our knowledge, are the highest values among all the CdS-based noble metal-free photocatalysts (Table S2†). From these results, it can be concluded that the loading of NiS can significantly improve the photocatalytic H_2_ evolution performance of CdS NWs with a much higher HER rate and more efficient conversion of visible light energy.

The stability and reusability of the obtained NiS/CdS NW hybrid photocatalysts were evaluated through six consecutive runs of the HER under the same conditions. Each cycle was performed under visible light irradiation (*λ* ≥ 420 nm) for 2 h. After each run, the reaction system was re-evacuated. Fig. 2d displays the recycling performance of the photocatalytic HER over the optimized NiS/CdS NWs. The result reveals that there was no significant decrease in the H_2_ evolution ability after each cycle. The optimized NiS/CdS NW hybrid photocatalyst could maintain a similar photocatalytic activity for more than 12 h, indicating an excellent stability of the present hybrid for the photocatalytic HER.

### 3.2 Composition, structure, and synthesis mechanism

**Fig. 3:**
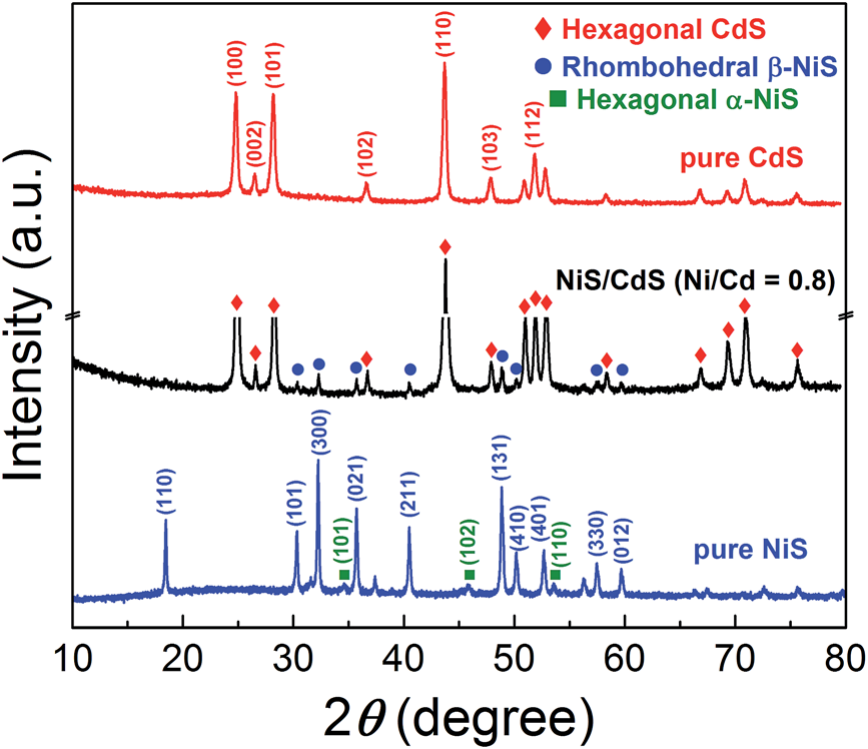
XRD patterns of the obtained pure CdS NWs, pure NiS nanostructures, and optimized NiS/CdS NWs.

In order to disclose the origin of the high photocatalytic HER efficiency observed for the hybrid NiS/CdS NWs, their compositions and structures were comprehensively investigated. Fig. 3 displays the XRD pattern of the optimized NiS/CdS NW hybrid photocatalyst, in comparison to that of the pure CdS NWs (Fig. S3†) and pure NiS nanostructures (Fig. S4†). For the pure CdS sample, the XRD pattern presents sharp diffraction peaks, indicating its good crystallinity. And all the diffraction peaks of this sample could be indexed to those of the hexagonal CdS phase (JCPDS no. 77-2306). For the pure NiS nanostructures, the main diffraction peaks matched well with those of the rhombohedral NiS phase (β-NiS, JCPDS no. 86-2281), while there were also some weak peaks (marked by green squares) with 2*θ* values of 34.67°, 45.91°, and 53.55° that can be assigned to the (101), (102), and (110) crystal planes, respectively, of the hexagonal NiS phase (α-NiS, JCPDS no. 75-0613). These results indicate that the pure NiS sample consists of β-NiS as the major phase and α-NiS as the minor phase. After NiS was loaded onto the CdS NWs, the major sharp diffraction peaks in the XRD pattern could be attributed to the CdS NWs (hexagonal, corresponding to JCPDS no. 77-2306), while several relatively weak peaks (marked by blue dots) could be observed at 2*θ* values of 30.31°, 32.21°, 35.71°, 40.47°, 48.84°, 50.14°, 57.43°, and 59.70°, matching well with the (101), (300), (021), (211), (131), (410), (330), and (012) crystal planes of β-NiS, respectively. In this case, no obvious peaks from α-NiS could be detected. This result is different from those reported previously in the literature about NiS/CdS photocatalysts, where the loaded NiS co-catalysts consisted of mainly α-NiS and a little of other phases such as β-NiS and nickel polysulfides (*e.g.* Ni_3_S_4_).^29^,^30^ As is known, compared to α-NiS, β-NiS has a smaller charge transfer resistance, which is beneficial for electronic transport through the material system, suggesting better activity for the HER.^35^ Thus, the presence of the almost pure β-NiS phase in the present NiS/CdS NW hybrid photocatalyst may be one of the most important reasons for its high visible light-driven HER activity.

**Fig. 4:**
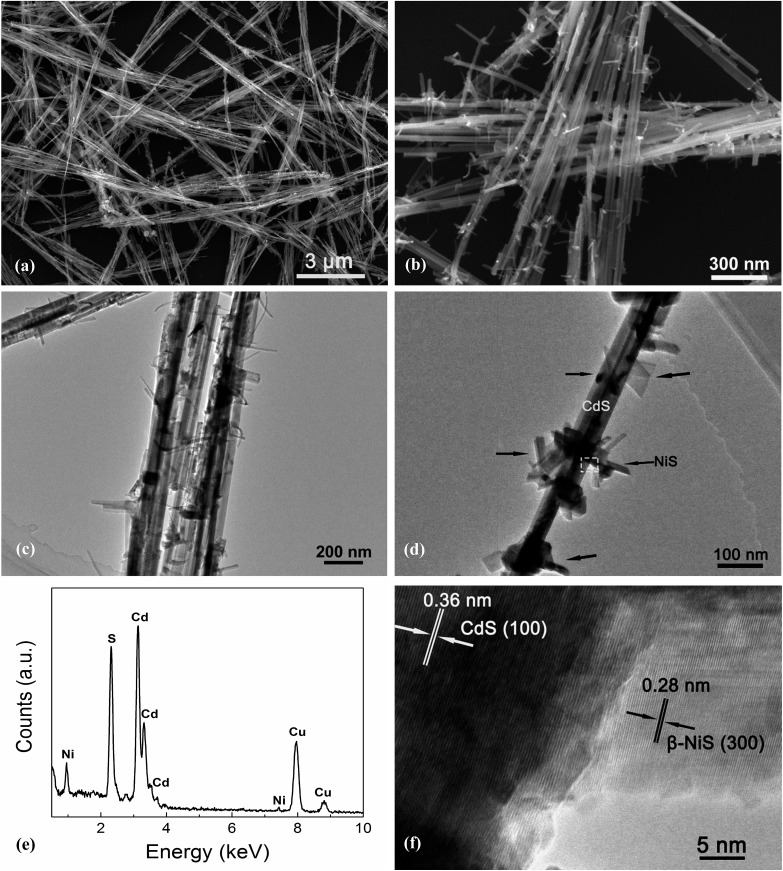
Microstructural characterization of the optimized NiS/CdS NWs. Typical low-magnification (a) and high-magnification (b) SEM images. Typical low-magnification (c) and high-magnification (d) TEM images. (e) Typical EDX spectrum. (f) HRTEM image corresponding to the marked square area in (d).

Fig. 4 exhibits the results of the microstructural characterization of the optimized NiS/CdS NWs. The SEM images shown in Fig. 4a and b reveal that the optimized NiS/CdS NWs have an average diameter of about 30 nm and a length of 5–10 μm. Such a high aspect ratio was inherited from the parent CdS NWs (Fig. S3†), implying that the synthesized NiS/CdS hybrid NWs would also have a large specific surface area as with the pure CdS NWs.^3^,^37^ Furthermore, there were no bulky clusters around the NWs; instead, some small flake-like branches could be observed on the surface of the obtained NWs, resulting in a larger contact area between NiS and the CdS NWs. By comparison with the SEM images of the pure NiS nanostructures (Fig. S4†), in which many flower-like nanosheets as well as some NPs could be observed, it can be deduced that the small flake-like branches on the surface of the obtained NWs might be β-NiS.

Further TEM observation (Fig. 4c and d) shows a consistent average diameter and length for the obtained NiS/CdS composite, and good dispersity of the flake-like branches on the surface of the CdS NWs. Besides, the EDX spectrum (Fig. 4e) of the optimized NiS/CdS NWs reveals the presence of Ni, S, Cd, and Cu, implying that the NiS co-catalyst was successfully loaded on the CdS NWs. As for Cu, the signal in the spectrum originated from the copper grid to support the TEM sample. More EDX examinations were also conducted at a single branch and the optimized NiS/CdS NWs, respectively, and the results are shown in Fig. S5.† These results confirm that the flake-like branches are indeed NiS, and the branches are well adhered to and densely dispersed onto the surface of the CdS NWs. To obtain more details about the contact area between the NiS flakes and CdS NWs, a HRTEM image corresponding to the square area shown in Fig. 4d is presented in Fig. 4f. Two distinct sets of lattice fringes can be identified from this image. The inter-planar spacing of 0.36 nm can be assigned to the (100) lattice plane of hexagonal CdS (JCPDS no. 77-2306), and that of 0.28 nm is attributed to the (300) plane of β-NiS (JCPDS no. 86-2281), matching well with the XRD results as discussed above, and indicating a good adhesion between the NiS flakes and CdS NWs. All these SEM and TEM results revealed that the highly reactive β-NiS co-catalyst was successfully loaded on the surface of the CdS NWs with a dense dispersion and intimate contact, which is beneficial for enhancing the HER efficiency of the synthesized hybrid photocatalysts.

To investigate why highly reactive β-NiS flakes, instead of other polymorphs, were formed, and why the β-NiS flakes were highly dispersed on and well adhered to the surface of the CdS NWs, several control experiments were conducted, with the aim of loading NiS onto the CdS NWs without the presence of NaH_2_PO_2_·H_2_O while keeping the other synthesis conditions fixed. The recorded XRD pattern (Fig. S6a†) shows that no NiS could be identified in the sample when the Ni/S FMR was fixed at 1 : 4 and the Ni/Cd FMR was at 0.8. Unexpectedly, a new weak peak could be identified at a 2*θ* value of 19.21°, which was indexed to hexagonal Ni(OH)_2_ (JCPDS no. 73-1520), indicating that a small amount of thiourea could not provide enough S to form NiS due to the slow decomposition rate of thiourea.^28^ And the formation of Ni(OH)_2_ might be attributed to the hydrolysis of Ni(CH_3_COO)_2_ under hydrothermal conditions, as demonstrated in ref. 40. However, when excessive thiourea (with a Ni/S FMR of 1 : 20, similar to the experiments in ref. 28–31) was used in the reaction but still without adding NaH_2_PO_2_·H_2_O, NiS_2_ and Ni_3_S_4_ were formed instead of NiS (Fig. S6b†). Such products were separately formed or aggregated together rather than being dispersed homogeneously onto the surface of the CdS NWs (Fig. S6c†), which would lead to poor contact between the co-catalysts and host photocatalysts. The photocatalytic activities of the samples prepared without NaH_2_PO_2_·H_2_O were also investigated (Fig. S6d†), both of which show activities lower than the one fabricated in the presence of NaH_2_PO_2_·H_2_O. According to these control experiments, it can be deduced that the applied NaH_2_PO_2_·H_2_O played a critical role in the synthesis of the hybrid NiS/CdS NWs.

Based on all the aforementioned discussions, the formation mechanism of the present NiS/CdS NW hybrid structure is proposed as follows (also see Fig. 1). When Ni(CH_3_COO)_2_ was added into the CdS NW suspension under magnetic stirring, the hydrolyzed Ni^2+^ could be adsorbed onto the CdS NWs to form a Ni^2+^/CdS precursor due to the presence of a lot of cation vacancies on the surface of the CdS NWs.^41^ At the early stage of the subsequent hydrothermal process, although the thiourea was not decomposed yet at low temperature, a small quantity of Ni^2+^ or Ni(CH_3_COO)_2_ had been already reduced into metallic Ni by H_2_PO_2_^–^*via* a process as follows:^42^

2 Ni^2+^ + 2H_2_PO_2_^–^ + 2H_2_O → Ni + 2H_2_PO_3_^–^ + 2H^+^ + H_2_

3 Ni(CH_3_COO)_2_ + 2H_2_PO_2_^–^ + 2H_2_O → Ni + 2H_2_PO_3_^–^ + 2H^+^ + H_2_ + 2CH_3_COO^–^,

forming a metallic Ni thin film on the surface of the CdS NWs. The formation of the metallic Ni film provided a crucial platform to induce the growth of highly dispersed NiS flake-like branches in the subsequent reaction. During the later hydrothermal process at 180 °C, the electroless plating process was ceased because of the high temperature, while the newly produced, highly active metallic Ni would react with thiourea to produce NiS, thus loading NiS on the surface of the CdS NWs continually.

In addition, in ref. 35, it had been proven that decreasing the Ni : S ratio in the precursor would facilitate the formation of highly reactive β-NiS. However, a small amount of thiourea cannot provide enough S to form NiS due to the slow decomposition rate of thiourea, as discussed above. When NaH_2_PO_2_·H_2_O is added in, H_2_PO_2_^–^ can accelerate the decomposition of thiourea,^42^ which would facilitate the formation of β-NiS even with a low concentration of thiourea under the present synthesis conditions. Consequently, almost pure β-NiS was formed and it was highly dispersed on and well adhered to the surface of the CdS NWs.

**Fig. 5:**
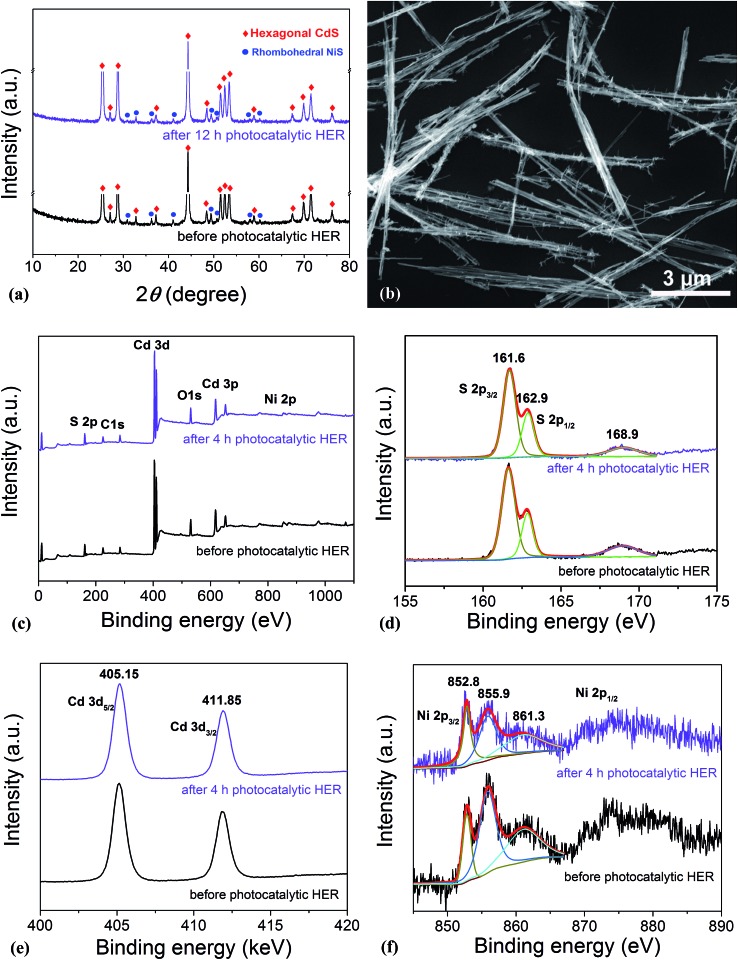
Investigation of the morphology, phase composition, and chemical stability of the optimized NiS/CdS NWs. (a) XRD patterns before and after 12 h of photocatalytic HER. (b) SEM image after 12 h of photocatalytic HER. (c) XPS survey spectra. (d), (e), and (f) are the S 2p, Cd 3d, and Ni 2p high-resolution spectra before and after 4 h of HER, respectively. The photocatalytic reaction conditions: 5 mg of the catalyst; 100 mL of aqueous solution containing 20 vol% lactic acid; visible light irradiation (*λ* ≥ 420 nm) provided by a 300 W Xe lamp with an UV cut-off filter; and 7 °C.

As is well known, stability and repeatability are very important aspects of a photocatalyst. Thus, the phase composition and morphology of the photocatalysts after a recycling photocatalytic HER were investigated, and the results are shown in Fig. 5a and b, respectively. These results reveal that there is no obvious change in both the morphology and phase composition of the photocatalyst after a 12 h consecutive reaction, which may be due to the good adhesion between NiS and CdS and the high crystallinity of the β-NiS. Besides, the chemical bonding states of the elements in the photocatalysts before and after the HER were also examined by XPS. As shown in Fig. 5c, the XPS survey spectra indicate the presence of Cd, S, Ni, O, and C in both samples. The carbon peak is attributed to the hydrocarbon in the XPS instrument itself. The two peaks for S 2p_3/2_ in the narrow S spectrum (Fig. 5d) at 161.6 and 162.9 eV are close to the binding energy of S^2–^ in CdS and NiS, respectively.^28^ The weak peak at 168.9 eV may be assigned to the small amount of S in a higher valence state, which came from the hydrolysis of thiourea. And the two peaks of Cd 3d (Fig. 5e) at 405.15 and 411.85 eV are attributed to the Cd 3d_5/2_ and Cd 3d_3/2_ in CdS, respectively.^23^ The high-resolution Ni 2p spectra are shown in Fig. 5f. The peak at 852.8 eV is close to the reported value for NiS,^43^ and the two peaks at 855.9 and 861.3 eV can be assigned to the main and satellite peaks of Ni^2+^ in Ni(OH)_2_,^27^ which came from the hydrolysis of Ni(CH_3_COO)_2_ under hydrothermal conditions, as discussed above. But Ni(OH)_2_ was not detected by XRD, SEM, and TEM characterizations in the samples, which might be due to the fact that Ni(OH)_2_ was of poor crystallinity and/or it was absorbed on the surface of the samples with an amorphous structure. Moreover, as compared in Fig. S6d,† the Ni(OH)_2_ modified CdS displays much lower photocatalytic HER activity than the optimized NiS/CdS sample, revealing that the high activity of the present hybrid photocatalysts is mainly derived from NiS instead of Ni(OH)_2_. In addition, after the 4 h photocatalytic HER, the peaks at 855.9 and 861.3 eV have markedly attenuated, indicating the decreased content of Ni(OH)_2_, which might result from its decomposition with acidic corrosion in lactic acid. However, there is no obvious attenuation in the peak at 852.8 eV, suggesting the good chemical stability of NiS during the photocatalytic HER. In addition, from Fig. 5d–f, no obvious changes in the binding energy of the Cd, S, and Ni elements can be observed before and after the 4 h photocatalytic HER, revealing that there is no valence change in these elements and implying that the chemical stability of the present photocatalyst is very high. In summary, the excellent stability in the morphology, composition, and surface chemistry contributes significantly to the outstanding photocatalytic HER activity of the present NiS/CdS NW hybrid photocatalyst.

### 3.3 Optoelectrochemical properties and photocatalytic HER mechanism

**Fig. 6:**
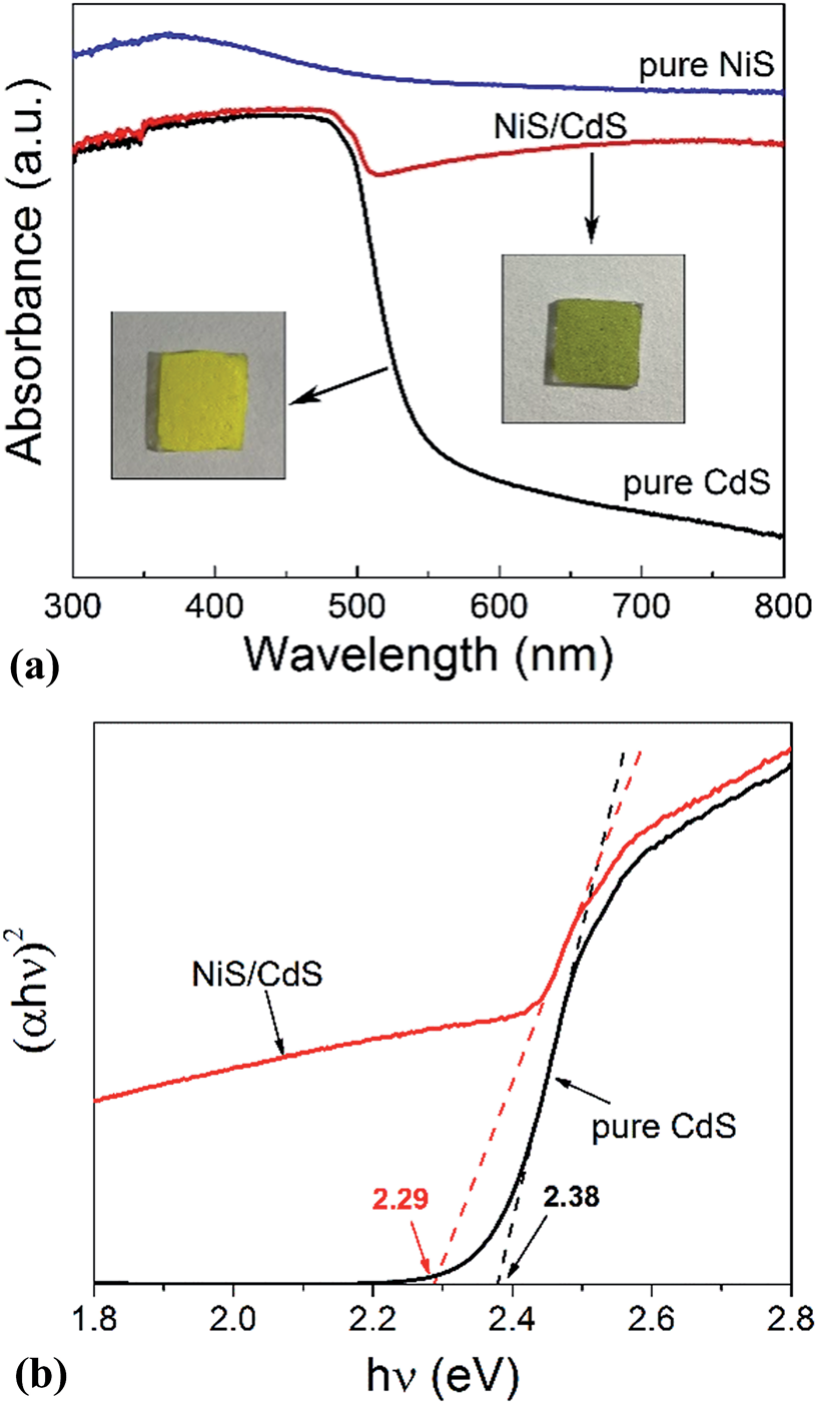
(a) UV-visible absorption spectra of the pure CdS NWs, pure NiS nanostructures, and optimized NiS/CdS NWs. The insets display the digital photographs of the corresponding samples, which were dip-coated and naturally dried onto 1 × 1 cm glass substrates. (b) The corresponding curves of (*αhν*)^2^*versus hν*.

In order to shed more light on the mechanism of the photocatalytic HER over the present NiS/CdS NW hybrid photocatalyst, the optoelectrochemical properties were further examined in comparison to those of the obtained pure CdS NWs and NiS nanostructures. Fig. 6a displays the UV-visible absorption spectra of pure CdS NWs, pure NiS nanostructures, and the optimized NiS/CdS NWs. As seen from this figure, the onset of the absorption edge of the pure CdS NWs is located at about 520 nm, which is in good agreement with the reported value in the literature.^28^–^30^ However, after β-NiS was loaded onto the CdS NWs, the absorption in the visible light region after 510 nm was substantially enhanced, which can be confirmed by the colour change of both samples (insets in Fig. 6a). These results indicate that the loading of the β-NiS co-catalyst can effectively broaden the region of light absorption. The enhanced light absorption can be attributed to the presence of the low band-gap black NiS in the NiS/CdS NW composite, which has a strong broad absorption in the range of 300–800 nm (Fig. 6a). In addition, a slight red-shift of the absorption edge compared to that of the pure CdS NWs could be observed, indicating that there is a decrease in the band-gap energy (*E*_g_) of the optimized NiS/CdS hybrid photocatalyst. Furthermore, the band-gaps of both samples were estimated from their Tauc plots as shown in Fig. 6b, *i.e.* the curves of (*αhν*)^*r*^*versus* photon energy (*hν*) derived from the UV-vis spectra, in which *r* = 2 because CdS is a direct band-gap semiconductor, by measuring the *x*-axis intercept of the extrapolated line from the linear region of the curve. The calculated *E*_g_ values of the pure CdS NWs and optimized NiS/CdS NWs were 2.38 and 2.29 eV, respectively. As expected, a decrease in *E*_g_ was determined, indicating that some Ni^2+^ ions might be doped into the CdS lattice. For the doping, the cation exchange reaction, Ni^2+^ + CdS → Cd^2+^ + NiS, is not a favorable route, because NiS has a much larger solubility than CdS.^28^ Instead, doping may happen through the formation of metallic Ni during the hydrothermal reaction as in the above-mentioned synthesis mechanism, which will give rise to the reaction of Ni^0^ + CdS → Cd^0^ + NiS.^44^

**Fig. 7:**
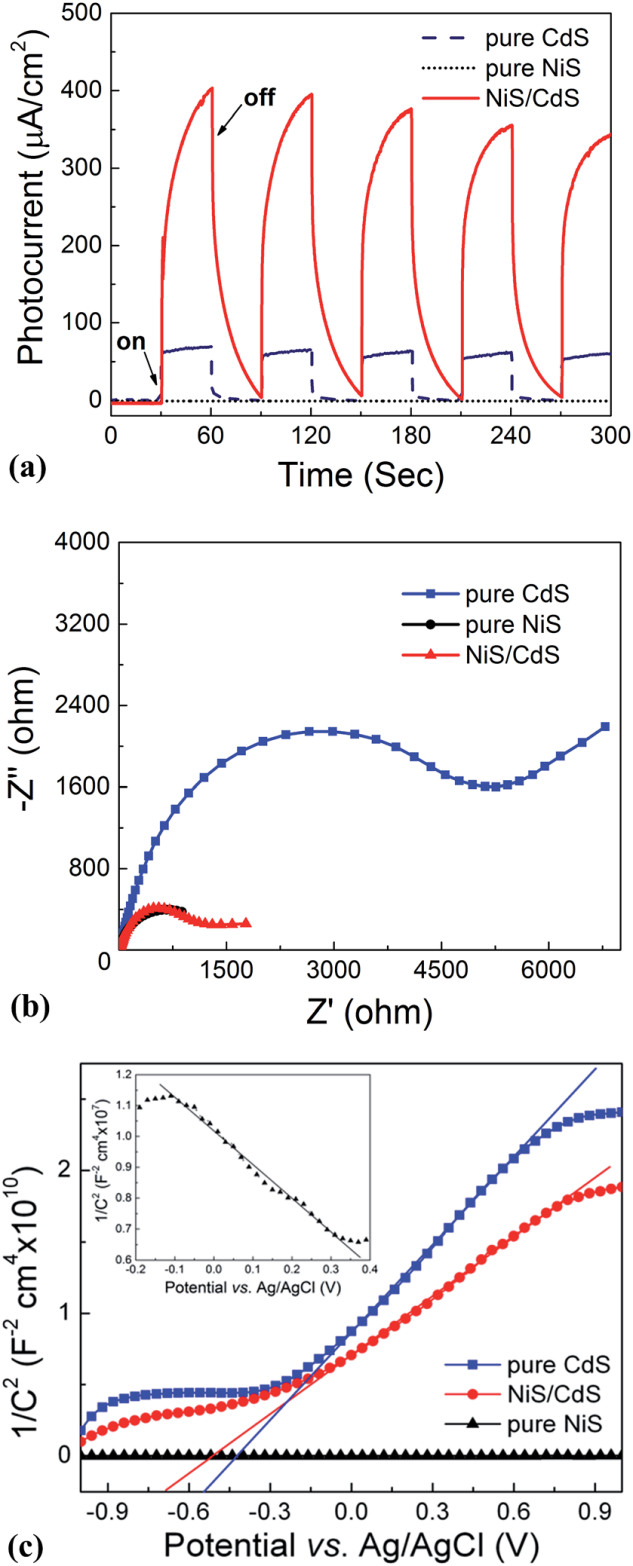
Photoelectrochemical measurements of the pure CdS NWs, pure NiS nanostructures, and optimized NiS/CdS NWs. (a) Transient photocurrent responses. (b) EIS Nyquist plots. (c) M–S plots, in which the inset is the zoomed view for the pure NiS nanostructures.

Moreover, the investigation on the transient photocurrent as shown in Fig. 7a reveals that, compared to the optimized NiS/CdS NWs, pure CdS NWs exhibit a weaker photocurrent, which could be attributed to the easy recombination of photo-generated electron–hole pairs in pure CdS. But when β-NiS was loaded onto the surface of the CdS NWs, the photocurrent of the samples was significantly enhanced. On the other hand, no obvious photocurrent was detected on the electrode coated by pure NiS nanostructures. These results reveal that the loaded β-NiS onto the CdS NWs has no significant contribution to the generation of photocarriers, in spite of its strong broad absorption in the range of 300–800 nm (Fig. 6a). Moreover, this conclusion is also supported by the IPCE measurements. As is seen in Fig. S7,† under light irradiation in the range of 400–520 nm, pure β-NiS nanostructures do not show any photo-to-electron conversion, while pure CdS NWs only present a low photon-to-electron conversion efficiency. However, after the loading of β-NiS onto the CdS NWs, the photon-to-electron conversion efficiency is dramatically enhanced. Meanwhile, under light irradiation above 520 nm, all three samples (pure CdS, pure NiS, and the NiS/CdS hybrid structure) have a very low photon-to-electron conversion efficiency. The aforementioned facts corroborate that in the present NiS/CdS NW hybrids, β-NiS is not a photocatalyst but only serves as a co-catalyst for CdS NWs. Such a co-catalyst would promote the separation of photo-generated electron–hole pairs and enhance the transfer efficiency of photo-generated carriers.

In addition, the EIS Nyquist plots (Fig. 7b) reveal that the impedance of pure CdS NWs is the highest, compared to the quite smaller values for the pure NiS nanostructures and the optimized NiS/CdS NWs, confirming the high charge transfer rate in the hybrid NiS/CdS NW sample after loading β-NiS onto the CdS NWs. This phenomenon should stem from the intimate contact between the loaded β-NiS and CdS NWs, and the high conductivity of β-NiS. All these results indicate that loading β-NiS onto CdS NWs is beneficial for the photocatalytic HER.

To further investigate the transfer of photo-generated charge carriers in the NiS/CdS NW hybrid structure, impedance spectroscopy was performed at a fixed AC frequency of 1 kHz to acquire the M–S plot. As shown in Fig. 7c, the pure CdS NWs present a positive slope, while the pure NiS nanostructures have a negative one (inset in Fig. 7c), indicating an n-type and p-type semiconductor behaviour for the two samples, respectively. But for the NiS/CdS NW hybrid structure, the M–S plot also shows a positive slope in the whole range without any p–n junction characteristic (*e.g.*, with a “V-shape” M–S plot), indicating that there is no p–n junction formed between the n-type CdS NWs and p-type β-NiS. As a result, the photo-generated electrons can be easily transferred from CdS to NiS. This is completely different from the previously reported NiS/CdS composites in the literature.^29^,^45^ And this phenomenon should be attributed to the electroless plating process during the formation of the NiS/CdS NW hybrid structure, as discussed above.

**Fig. 8:**
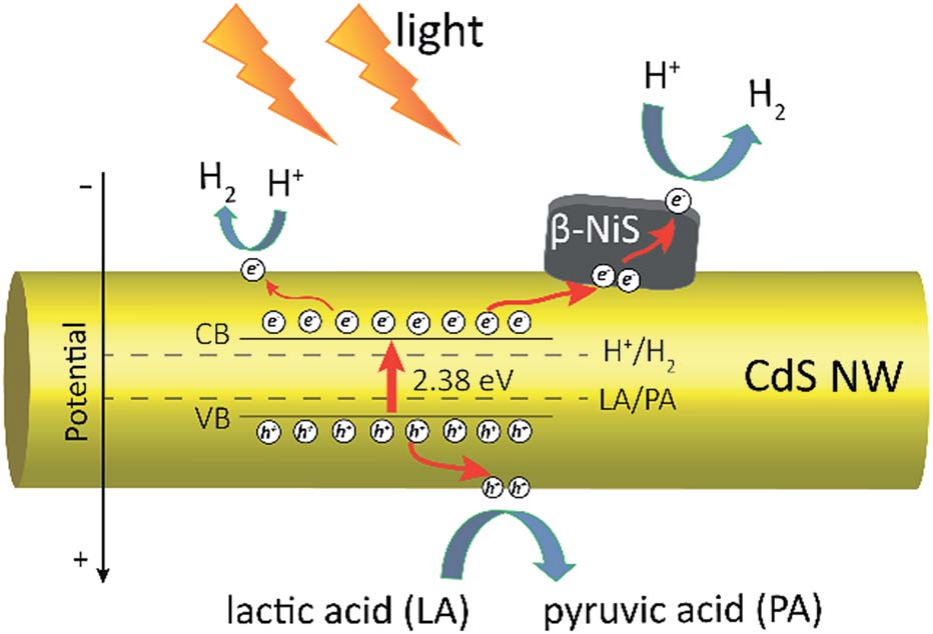
Schematic of the photocatalytic HER mechanism over the obtained NiS/CdS NWs.

Based on all the above observations and discussions, the photocatalytic HER mechanism over the hybrid NiS/CdS NWs can be depicted as in Fig. 8. As can be seen, under visible light irradiation, the photo-generated electrons (e^–^) jump into the CB of CdS, leaving holes (h^+^) in the VB. The photo-generated electrons can partially move toward the surface of CdS, directly reducing H^+^ in the solution from water or lactic acid to H_2_. Because no p–n junction exists in the present NiS/CdS hybrid structure, the photo-generated electrons can be easily transferred to the surface of β-NiS due to the intimate contact between the host CdS and co-catalyst β-NiS and high electrical conductivity of β-NiS. As a result, the separation of photo-generated electron–hole pairs is substantially improved. Subsequently, the electrons on the surface of β-NiS can reduce the H^+^ in the solution to H_2_ effectively due to the high electrocatalytic HER activity of β-NiS. This process can be illustrated in eqn (4)–(6):^28^4
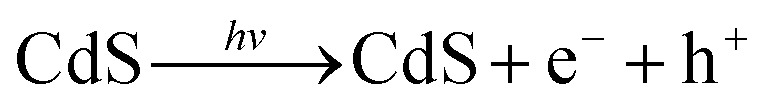

5 NiS + H^+^ + e^–^ → HNiS

6 HNiS + H^+^ + e^–^ → NiS + H_2_

In this case, the highly electrocatalytically active β-NiS would also serve as the active sites for the photocatalytic HER. In other words, the reduction of H^+^ to H_2_ can also proceed on the surface of β-NiS, thus maximizing the photocatalytic HER activity of the present NiS–CdS composite catalyst. At the same time, the photo-generated h^+^ can be absorbed effectively by lactic acid on the surface of CdS, which can oxidize the lactic acid to pyruvic acid. And such a process has been proven to be the only pathway for the consumption of photo-generated holes.^28^ Moreover, the presence of the β-NiS co-catalyst also enhanced the visible light-harvesting ability of the NiS/CdS hybrid catalyst (Fig. 6a), which might be another reason for the enhanced photocatalytic activity. In summary, the dramatically enhanced photocatalytic HER activity of the NiS/CdS hybrid catalyst can be attributed to the synergistic effect of the enhanced visible light-harvesting ability and highly effective separation of photo-generated electron–hole pairs, resulting from the loading of the β-NiS co-catalyst.

In addition, for many photocatalysts based on CdS,^3^,^21^,^23^ Na_2_SO_3_ and Na_2_S could be used as the sacrificial agents, presenting high HER activities. However, for the photocatalytic system over the present NiS/CdS NW hybrid catalyst, the HER activities in Na_2_SO_3_ and Na_2_S solutions were much lower than that in lactic acid aqueous solution (Fig. S1†). It seems that the acidic conditions may facilitate the photocatalytic HER over the hybrid NiS/CdS NWs, possibly because the high concentration of H^+^ in the acidic solution could promote the reactions of eqn (5) and (6).

## 4 Conclusions

A β-NiS modified CdS NW hybrid structure was synthesized *via* a novel hydrothermal synthesis method. The as-obtained NiS/CdS NWs show high photocatalytic HER activity under visible light irradiation. The HER rate measured at 7 °C over the optimal NiS/CdS NWs prepared at a Ni/Cd feed molar ratio of 0.8 was 592.1 μmol h^–1^, and the AQY at 420 nm was 57.8%. When the reaction temperature was increased to 25 °C, they could be further improved up to 793.6 μmol h^–1^ and 74.1%, respectively. To the best of our knowledge, the present system exhibits the highest AQY among all the CdS-based noble metal-free photocatalysts.

In the present synthesis route, NaH_2_PO_2_·H_2_O plays a critical role in achieving such highly active NiS/CdS NW hybrid photocatalysts. It is proposed that a metallic Ni film intermediate is first formed *via* an electroless plating process assisted by H_2_PO_2_^–^, which would then induce the growth of β-NiS flake-like branches on the surface of the CdS NWs with high dispersion and intimate contact. Meanwhile, H_2_PO_2_^–^ could also accelerate the decomposition of thiourea, further facilitating the formation of highly active β-NiS at a low concentration of thiourea.

The mechanism for the photocatalytic HER over the present NiS/CdS NWs is also proposed. During the photocatalytic HER, the photo-generated electrons can be easily transferred to the surface of β-NiS due to the intimate contact between β-NiS and CdS and the high electrical conductivity of β-NiS, thus promoting the separation of photo-generated electron–hole pairs effectively. Besides, the presence of the β-NiS co-catalyst can enhance the visible light-harvesting ability of the NiS/CdS hybrid catalyst.

The present synthesis strategy provides new insights into the design and development of high-performance heterostructured photocatalysts for solar-driven H_2_ evolution.

## Conflicts of interest

There are no conflicts to declare.

## Supplementary Material

Supplementary information
