# Supplementary material for: β-NiS modified CdS nanowires for photocatalytic H2 evolution with exceptionally high efficiency
Source: Chem Sci. 2017 Dec 13;9(6):1574–85. doi: 10.1039/c7sc03928j (PMC5890328; doi:10.1039/c7sc03928j)
Supplement: Supplementary file 1 [file SC-009-C7SC03928J-s001.pdf]

Electronic Supplementary Material (ESI) for Chemical Science.
This journal is © The Royal Society of Chemistry 2017

Electronic Supplementary Information

β-NiS modified CdS nanowires for photocatalytic H_2 evolution with exceptionally high efficiency

Shundong Guan,[a,b] Xiuli Fu,*[a] Yu Zhang[a,b] and Zhijian Peng*[b]

[a] *State Key Laboratory of Information Photonics and Optical Communications, and School of Science, Beijing University of Posts and Telecommunications, Beijing 100876, P.R. China*

[b] *School of Science, China University of Geosciences, Beijing 100083, P.R. China*

[a]*xiulifu@bupt.edu.cn;* [b]*pengzhijian@cugb.edu.cn*

Financial support from the National Natural Science Foundation of China (grant nos. 11674035, 11274052, and 61274015), and Fund of State Key Laboratory of Information Photonics and Optical Communications (Beijing University of Posts and Telecommunications) is gratefully acknowledged.

Shundong Guan, et al., *Chemical Science*, Figure S1

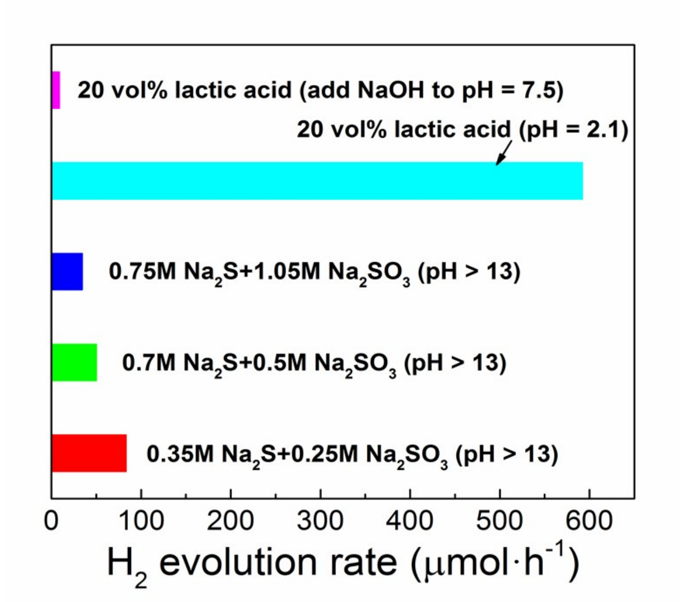

Figure S1 The H_2 evolution rate under different sacrificial agent solutions over the optimal NiS/CdS NWs prepared at the Ni/Cd feed molar ratio of 0.8. The data were calculated based on the H_2 amount generated in the first 4 h reaction. Reaction conditions: 5 mg of the catalyst; 100 mL of aqueous solution; 7 °C; and visible light irradiation ($\lambda \geq 420$ nm) provided by a 300 W Xe lamp with an UV cut-off filter. This figure reveals that the acidic conditions provided by 20 vol.% of lactic acid aqueous solution is beneficial for the photocatalytic H_2 evolution over the present NiS/CdS NWs.

Shundong Guan, et al., *Chemical Science*, Figure S2

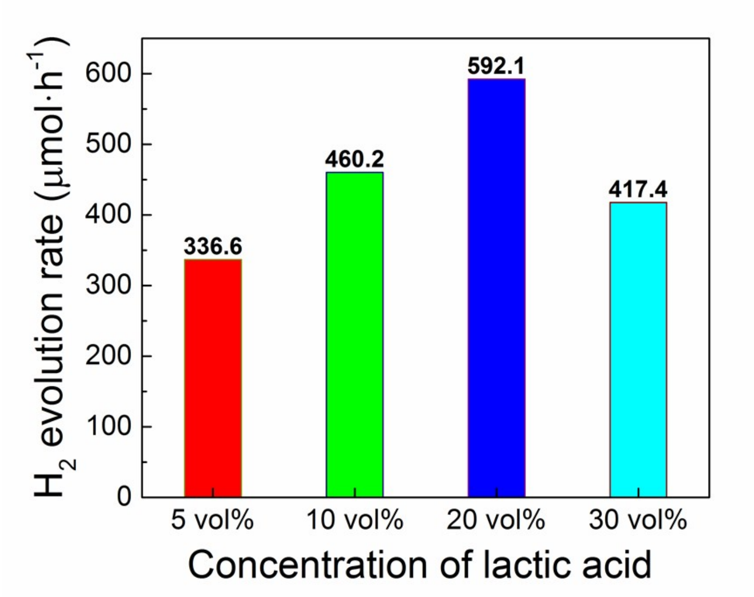

Figure S2 The rate of photocatalytic H_2 evolution in different concentrations of lactic acid aqueous solution over the β-NiS modified CdS nanowires (NiS/CdS NWs) prepared at a Ni/Cd feed molar ratio of 0.8 in the synthesis reaction. The data were calculated based on the H_2 amount generated in the first 4 h of reaction. Reaction conditions: 5 mg of the photocatalyst; 100 mL of aqueous solution; 7 ºC; and visible light irradiation ($\lambda \geq 420$ nm) provided by a 300 W Xe lamp with an UV cut-off filter. This figure reveals that the optimum concentration of lactic acid for the photocatalytic H_2 evolution over the as-prepared NiS/CdS NWs is 20 vol.%.

Shundong Guan, et al., *Chemical Science*, Figure S3

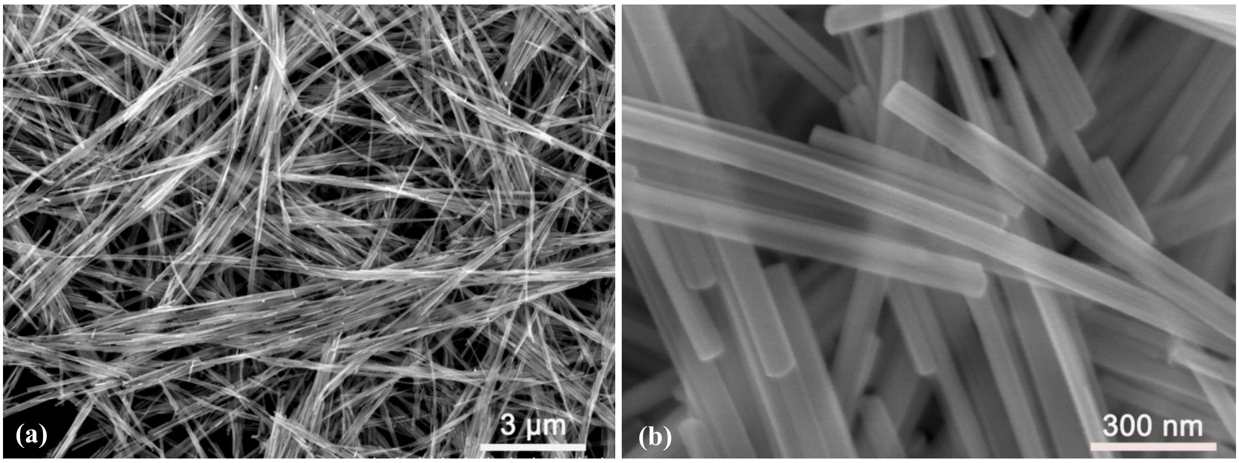

Figure S3 Typical SEM images of the synthesized pure CdS NWs at low (a) and high (b) magnifications. The CdS NWs have an average diameter of about 30 nm and a length of 5-10 μm.

Shundong Guan, et al., *Chemical Science*, Figure S4

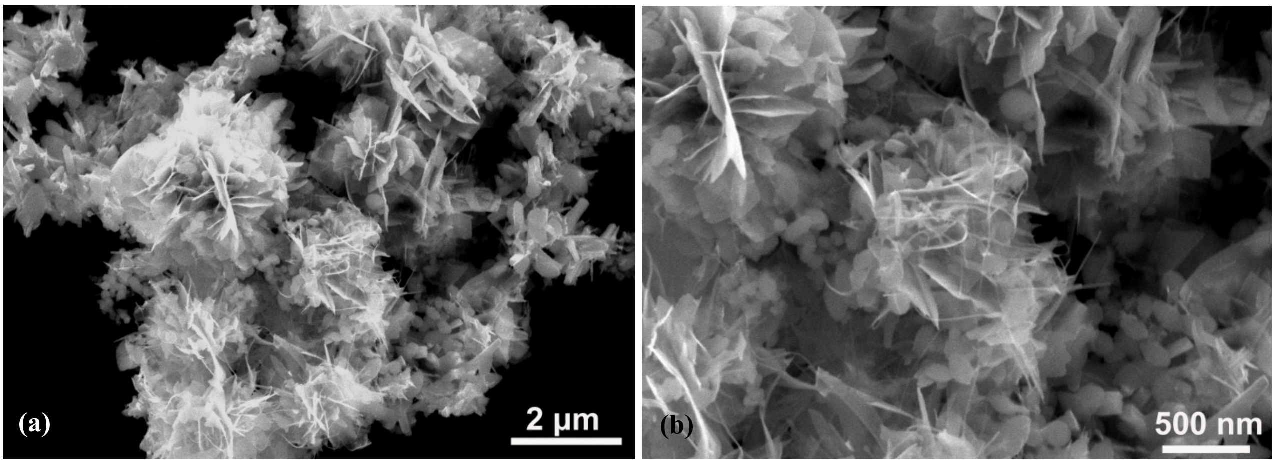

Figure S4 Typical SEM images of the synthesized pure NiS sample at low (a) and high (b) magnifications, indicating that it consists of flower-like nanosheets and nanoparticles.

Shundong Guan, et al., *Chemical Science*, Figure S5

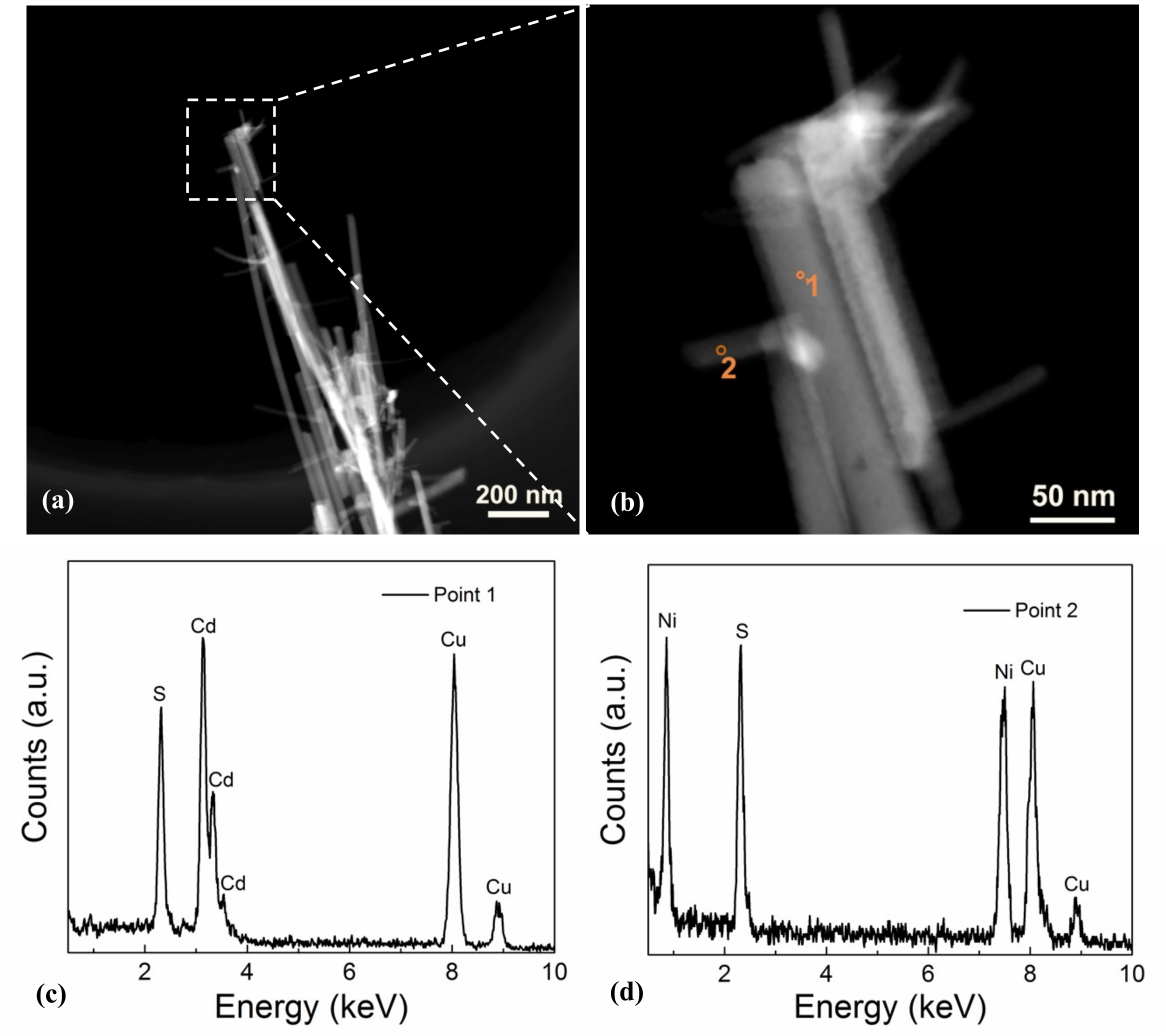

Figure S5 (a) low and (b) high magnification STEM images of the optimal NiS/CdS NWs prepared at the Ni/Cd feed molar ratio of 0.8. (c) and (d) EDX spectra of the point 1 and 2 as shown in (b), respectively. This figure indicates the successful loading of NiS nanoflakes onto CdS NWs.

Shundong Guan, et al., *Chemical Science*, Figure S6

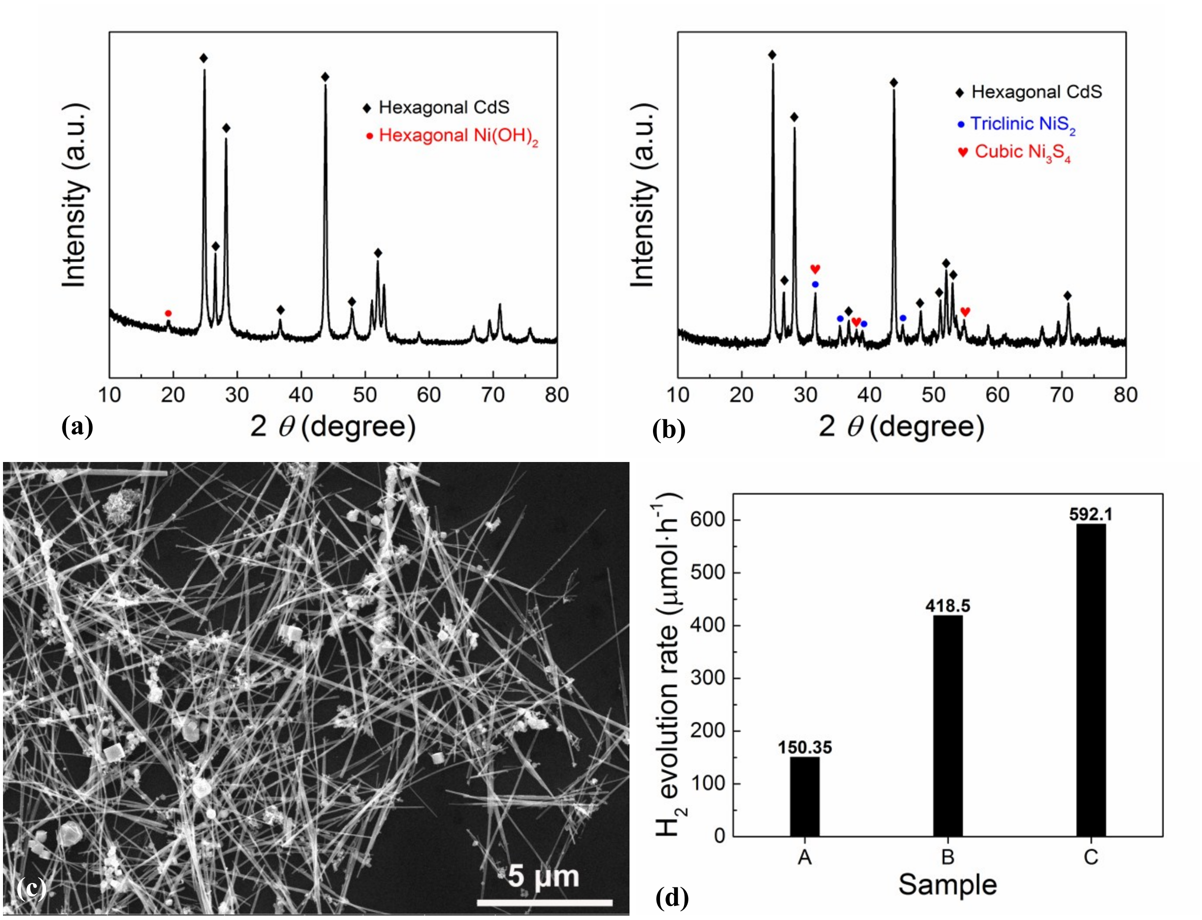

Figure S6 XRD patterns of the products prepared at a Ni/Cd feed molar ratio of 0.8 without $NaH_2PO_2 \cdot H_2O$ while the Ni/S molar ratio was 1:4 (a) and 1:20 (b), respectively. (c) Typical SEM image of the product prepared at a Ni/Cd feed molar ratio of 0.8 and Ni/S feed molar ratio of 1:20 without $NaH_2PO_2 \cdot H_2O$. (d) The H_2 evolution rate over the products prepared under different conditions. Among them, Samples A and B were prepared at a Ni/Cd feed molar ratio of 0.8 without $NaH_2PO_2 \cdot H_2O$ while the Ni/S molar ratio was 1:4 and 1:20, respectively; and Sample C was prepared at a Ni/Cd feed molar ratio of 0.8 with 0.6 mmol of $NaH_2PO_2 \cdot H_2O$ while the Ni/S feed molar ratio was 1:4. The data were calculated based on the H_2 amount generated in the first 4 h reaction. Reaction conditions: 5 mg of the catalysts; 100 mL of aqueous solution containing 20 vol.% lactic acid; 7 °C; and visible light irradiation ($\lambda \geq 420$ nm) provided by a 300 W Xe lamp with a cut-off filter. This figure reveals that $NaH_2PO_2 \cdot H_2O$ is crucial for the successful loading of β-NiS onto CdS NWs to prepare a high-performance photocatalyst.

Shundong Guan, et al., *Chemical Science*, Figure S7

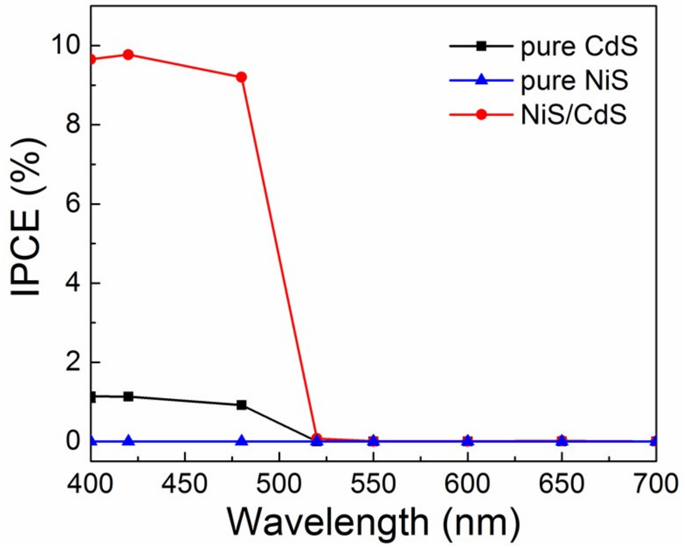

Figure S7 Incident photon-to-electron conversion efficiencies (IPCE) of the pure CdS NWs, pure NiS nanostructures and optimal NiS/CdS NWs prepared at the Ni/Cd feed molar ratio of 0.8.

All the IPCE data were calculated by,[S1]

$$IPCE = \frac{1240 \times J}{\lambda \times I_{light}} \times 100\% \qquad (1)$$

where J is the measured photocurrent, and I_{light} is the light intensity at the wavelength λ of 400±5, 420±5, 480±5, 520±5, 550±5, 600±5, 650±5 and 700±5 nm, respectively, which is measured by an irradiatometer (FZ-A, Photoelectric Instrument Factory of Beijing Normal University, Beijing, China).

As is seen from this figure, under light irradiation in the range of 400-520 nm, pure β-NiS nanostructures almost have no ability to photo-to-electron conversion, while pure CdS NWs presented a small photon-to-electron conversion efficiency. However, after the loading of β-NiS onto CdS NWs, the photon-to-electron conversion efficiency was dramatically enhanced through the present NiS/CdS NWs hybrid structures. Meanwhile, under light irradiation after 520 nm, all the three samples (pure CdS, pure NiS and NiS/CdS hybrid structure) have very low photon-to-electron conversion efficiency. These results reveal that in the present NiS/CdS NWs hybrids, NiS is not a photocatalyst but only serves as a co-catalyst for CdS NWs to effectively promote the separation of photo-generated electron-hole pairs.

Shundong Guan, et al., *Chemical Science*, Table S1

Table S1 Practical molar ratios of NiS in the photocatalysts prepared at various Ni/Cd feed molar ratios measured by SEM-EDX analysis

**Designed Ni/Cd feed molar ratio**	0:1 (pure CdS)	0.2:1	0.3:1	0.4:1	0.5:1	0.6:1
**Practical molar percentage of NiS**	0%	0.53%	0.96%	2.39%	4.87%	7.01%
**Designed Ni/Cd feed molar ratio**	0.7:1	0.8:1	0.9:1	1:1	1.2:1	1:0
**Practical molar percentage of NiS**	9.89%	11.75 %	13.8%	15.2%	18.6%	100% (pure NiS)

Table S2 Review on CdS-based noble metal-free photocatalysts for H_2 evolution

Photocatalyst	Co-catalyst	Light source[a]	Aqueous[b]	T[c]	H_2 evolution			Ref.
					Rate ($\mu mol \cdot h^{-1} \cdot g^{-1}$)	AQY (%)	F[d]	
CdS nanowires	NiS	300 W Xe ($\lambda \geq 420$ nm)	20 vol% Lactic acid	7 ˚C	118420	57.8 (420 nm)	204	This work
				25 ˚C	158720	74.1 (420 nm)	250	
CdS nanorods	0.5 wt% Ni_2P	300 W Xe ($\lambda \geq 420$ nm)	1.05 M Na_2SO_3 + 0.75 M Na_2S	RT	1200000	41 (450 nm)	22	[3]
CdS nanorods	Ni	LED (447 nm)	Ethanol	RT	63000	53 (447 nm)		[5]
CdS	MoP	300 W Xe ($\lambda \geq 420$ nm)	20 vol% Lactic acid	20 ˚C	73333	45 (460 nm)		[11]
CdS	15 wt% MoS_2	300 W Xe ($\lambda \geq 420$ nm)	0.02 M Na_2SO_3 + 0.1 M Na_2S	RT	4470		10	[12]
CdS	0.2 wt% MoS_2	300 W Xe ($\lambda \geq 420$ nm)	10 vol% Lactic acid	RT	5400		36	[13]
CdS nanorods	16.7 wt% MoP	300 W Xe ($\lambda \geq 420$ nm)	10 vol% Lactic acid	RT	163200	5.8 (450 nm)	20	[14]
CdS	11 mol% WS_2	300 W Xe ($\lambda \geq 420$ nm)	10 vol% Lactic acid	RT	1984		16	[15]
CdS	10 wt% WC	500 W Xe ($\lambda \geq 420$ nm)	0.02 M Na_2SO_3 + 0.1 M Na_2S	RT	1400		23	[16]
CdS	1 wt% WS_2	300 W Xe ($\lambda \geq 420$ nm)	10 vol% Lactic acid	10±5 ˚C	4200	5 (420 nm)	28	[17]
CdS nanorods	6 wt% Co_2P	300 W Xe	10 vol% Lactic acid	RT	10800		16	[18]
CdS nanowires	6.5 mol% $Co(OH)_2$	300 W Xe ($\lambda \geq 420$ nm)	30 vol% TEOA	RT	14430		206	[19]
CdS nanorods	6.8 mol% $Co(OH)_2$	500 W Xe	25 vol% Ethanol	RT	61		41	[20]
CdS nanorods	3 mol% Co_3O_4	300 W Xe ($\lambda \geq 420$ nm)	0.5 M Na_2SO_3 + 0.5 M Na_2S	RT	236		33	[21]
CdS nanorods	4.86 wt% Ni_3N	300 W Xe ($\lambda \geq 420$ nm)	0.35 M Na_2SO_3 + 0.25 M Na_2S	RT	88000	~13 (420 nm)	10	[22]
CdS	13.2 mol% Ni_2O_3	300 W Xe ($\lambda \geq 400$ nm)	30 vol% Methanol	RT	4456		4.1	[23]
CdS	1 mol% NiO_x	300 W Xe ($\lambda \geq 400$ nm)	30 vol% Methanol	RT	5908	8.6 (400 nm)	117	[24]
CdS	32 mol% NiO	500 W Phoenix tungsten halogen lamp	0.25 M Na_2SO_3 + 0.35 M Na_2S	RT	745	6.02		[25]

CdS nanorods	23 mol% $Ni(OH)_2$	300 W Xe (λ ≥ 420 nm)	25 vol% TEOA	RT	5084	28 (420 nm)	145	[26]
CdS	1.2 mol% NiS	300 W Xe (λ ≥ 420 nm)	30 vol% Lactic acid	RT	7267	51.3 (420 nm)	34	[27]
CdS nanorods	5 mol% NiS	300 W Xe (λ ≥ 420 nm)	0.25 M Na_2SO_3 + 0.35 M Na_2S	RT	1131	6.1 (420 nm)	21	[28]
CdS	NiS	300 W Xe (λ ≥ 420 nm)	30 vol% Lactic acid	35 ˚C	28600	60.4 (420 nm)	~30	[29]
CdS nanorods	3 mol% CuS	500 W Xe	0.25 M Na_2SO_3 + 0.35 M Na_2S	RT	332		3.5	[31]
CdS nanorods	0.44 wt% Cu_3P	300 W Xe (λ ≥ 420 nm)	1.75 M Na_2SO_3 + 1.25 M Na_2S	RT	200000	25 (420 nm)	6.6	[32]
CdS nanorods	4 wt% Ni	300 W Xe (λ ≥ 420 nm)	1 M $(NH_4)_2SO_3$	RT	25848	26.8 (420 nm)		[S2]
CdS nanorods	5 wt% Ni	300 W Xe (λ ≥ 400 nm)	50 vol% Lactic acid	RT	30048			[S3]
CdS	Ni	300 W Xe (λ ≥ 395 nm)	4.6 M Na_2SO_3 + 3.3 M Na_2S	RT	2.5		10	[S4]
CdS	0.4 wt% RGO +2 wt% MoS_2	500 W UV-vis lamp	10 vol% Lactic acid	RT	6857		71	[S5]
CdS nanorods	30 wt% Fe_2P	300 W Xe (λ ≥ 420 nm)	0.5 M Ascorbic acid	RT	186000	15 (450 nm)	31	[S6]
CdS	2 wt% Ni_2P	simulated solar radiation	20 vol% Lactic acid	RT	33480		62	[S7]
CdS nanorods	6 wt% WS_2-MoS_2	150 W Xe (AM 1.5G)	20 vol% Lactic acid	RT	209790	51.4 (425 nm)	83	[S8]
CdS nanowires	MoS_2	300 W Xe (λ ≥ 400 nm)	50 vol% Lactic acid	5 ˚C	10850	22 (475 nm)	28	[S9]
CdS	MoS_2	300 W Xe (λ ≥ 420 nm)	20 vol% Lactic acid	RT	3875	14.7 (420 nm)	65	[S10]
CdS nanorods	5.0 wt% Cu_2MoS_4	150 W Xe	20 vol% Lactic acid	RT	15560		4.2	[S11]
CdS	Ti_3C_2	300 W Xe (λ ≥ 420 nm)	22 vol% Lactic acid	RT	14342	40.1 (420 nm)	136	[S12]
CdS nanorods	6 wt% MoS_2	natural solar radiation	20 vol% Lactic acid	RT	174000	38.7 (425 nm)	14.5	[S13]

a) Xe: xenon lamp; Hg: mercury lamp

b) TEOA: triethanolamine

c) T: reaction temperature; RT: room temperature

d) F: enhancement factor (vs. pure CdS)

References

[S1] F.K. Meng, J.T. Li, Scott K Cushing, M.J. Zhi, N. Q. (Nick) Wu. Solar Hydrogen Generation by Nanoscale p-n Junction of p-type Molybdenum Disulfide/n-type Nitrogen-Doped Reduced Graphene Oxide. Journal of American Chemical Society, 2013, 135 (28): 10286–10289.

[S2] H. Wang, W. Chen, J. Zhang, C.P. Huang, L.Q. Mao. Nickel nanoparticles modified CdS: A potential photocatalyst for hydrogen production through water splitting under visible light irradiation. International Journal of Hydrogen Energy, 2015, 40(1): 340-345.

[S3] S. Chen, X.P. Chen, Q.Z. Jiang, J. Yuan, C.F. Lin, W.F. Shangguan. Promotion effect of nickel loaded on CdS for photocatalytic H_2 production in lactic acid solution. Applied Surface Science, 2014, 316: 590-594.

[S4] S. Devi, P. Korake, S.N. Achary, N.M. Gupta. Genesis of enhanced photoactivity of CdS/Ni_x nanocomposites for visible-light-driven splitting of water. International Journal of Hydrogen Energy, 2014, 39(34): 19424-19433.

[S5] T.T. Jia, A. Kolpin, C.S. Ma, R.C.T. Chan, W.M. Kwok, S.C.E. Tsang. A graphene dispersed CdS-MoS_2 nanocrystal ensemble for cooperative photocatalytic hydrogen production from water. Chemical Communications, 2014, 50: 1185-1188.

[S6] Z.J. Sun, H.L. Chen, Q. Huang, P.W. Du. Enhanced photocatalytic hydrogen production in water under visible light using noble-metal-free ferrous phosphide as an active cocatalyst. Catalysis Science & Technology, 2015, 5: 4964-4967.

[S7] D.P. Kumar, J.H. Choi, S.Y. Hong, D.A. Reddy, S.H. Lee, T.K. Kim. Rational synthesis of metal-organic framework-derived noble metal-free nickel phosphide nanoparticles as a highly efficient cocatalyst for photocatalytic hydrogen evolution. ACS Sustainable Chemistry & Engineering, 2016, 4(12): 7158-7166.

[S8] D.A. Reddy, H. Park, R. Ma, D.P. Kumar, M.H. Lim, T.K. Kim. Heterostructured WS_2-MoS_2 ultrathin nanosheets integrated on CdS nanorods for promoting charge separation and migration to improve solar-driven photocatalytic hydrogen evolution. ChemSusChem, 2017, 10(7): 1563-1570.

[S9] H.F. Lin, Y.Y. Li, H.Y. Li, X. Wang. Multi-node CdS hetero-nanowires grown with defect-rich oxygen-doped MoS_2 ultrathin nanosheets for efficient visible-light photocatalytic H_2 evolution. Nano Research, 2017, 10(4): 1377-1392.

[S10] A.P. Wu, C.G. Tian, Y.Q. Jiao, Q. Yan, G.Y. Yang, H.G. Fu. Sequential two-step hydrothermal growth of MoS_2/CdS core-shell heterojunctions for efficient visible light-driven photocatalytic H_2 evolution. Applied Catalysis B: Environmental, 2017, 203: 955-963.

[S11] S.Y. Hong, D. Praveen Kumar, D.A. Reddy, J.H. Choi, T.K. Kim. Excellent photocatalytic hydrogen production over CdS nanorods via using noble metal-free copper molybdenum sulfide (Cu_2MoS_4) nanosheets as co-catalysts. Applied Surface Science, 2017, 396: 421-429.

[S12] J.R. Ran, G.P. Gao, F.T. Li, T.Y. Ma, A.J. Du, S.Z. Qiao. Ti_3C_2 MXene co-catalyst on metal sulfide photo-absorbers for enhanced visible-light photocatalytic hydrogen production. Nature Communications, 2017, 8: 13907.

[S13] D.P. Kumar, S.Y. Hong, D.A. Reddy, T.K. Kim. Noble metal-free ultrathin MoS_2 nanosheet-decorated CdS nanorods as an efficient photocatalyst for spectacular hydrogen evolution under solar light irradiation. Journal of Materials Chemistry A, 2016, 4: 18551-18558.
